# Supplementary material for: Evaluating the Efficacy of Plant Extracts in Managing the Bruchid Beetle, Callosobruchus maculatus (Coleoptera: Bruchidae)
Source: Insects. 2024 Sep 12;15(9):691. doi: 10.3390/insects15090691 (PMC11432110; doi:10.3390/insects15090691)
Supplement: Supplementary file 1 [file insects-15-00691-s001.zip › insects-3143343-supplementary.pdf]

Table S1. List of plant species and plant parts used in the experiment with *C. maculatus*

Sr. No.	CommonName	Botanical Name	Family	Part used
1	Neem	*Azadirachta indica*	Meliaceae	Seed
2	Bakion	*Melia azedarach*	Meliaceae	Fruit
3	WhitePatta	*Nicotiana rustica*	Solanaceae	Leave
4	Virginiatobacco	*Nicotiana tabacum*	Solanaceae	Leave
5	Chinesearborvitae	*Thuja orientalis*	Cupressaceae	Fruit

Table S2. Mean percent of *C. maculatus* oviposition (eggs/female)

Source		DF	SS	MS		F		P	
Replication	3	37.7	12.57						
Concentrations	5	11287.0		2257.40		1032.02		0.0000	
Plants		4	16580.5		4145.13		1895.04		0.0000
Concentrations *Plants		20	246.2	12.31		5.63		0.0000	
Error		87	190.3		2.19				
Total		119	28341.7						

Grand Mean 104.95 CV 1.41

Table S3. Mean percent of *C. maculatus s* adult emergence

Source		DF	SS	MS	F		P
Replications		3	9.3		3.12		
Plants		4	8675.0	2168.75		778.42	0.0000
Concentrations		5	6543.7	1308.73		469.74	0.0000
Plants* concentrations	20	331.5	16.57	5.95		0.0000	
Error		87	242.4		2.79		
Total		119	15801.9				

Grand Mean 69.086 CV 2.42

Table S4. Mean percent of host infestation due *C. maculatus*

Source		DF	SS	MS		F		P	
Replications		3	2.62	0.872					
Plants		4	1184.43		296.108		1344.56		0.0000
Concentrations		5	1699.28	339.856		1543.21		0.0000	
Plants*concentrations	20	11.74	0.587		2.67		0.0009		
Error		87	19.16	0.220					
Total		119	2917.23						

Grand Mean 21.300 CV 2.20

Table S5. Mean percent seed weight loss due to *C. maculatus* infestation.

Source	DF	SS	MS	F	P
Replications	3	2.17	0.72		
Plants	4	4836.38	1209.09	984.83	0.0000
Concentrations	5	1955.19	391.04	318.51	0.0000
Plants*concentrations 20	38.84	1.94	1.58	0.0758	
Error	87	106.81	1.23		
Total	119	6939.38			

Grand Mean 19.156 CV 5.78

Table S6. Mean percent *C. maculatus* male ratio.

Source	DF	SS	MS	F	P
Replications	3	2.0799	0.69329		
Plants	4	0.5826	0.14564	0.49	0.7428
Concentrations	5	0.9600	0.19201	0.65	0.6649
Plants* concentrations 20	1.7927	0.08963	0.30	0.9982	
Error	87	25.8421	0.29704		
Total 119 31.2572					

Grand Mean 50.094 CV 1.09

Table S7. Mean percent *C. maculatus* female male ratio.

Source	DF	SS	MS	F	P
Replications	3	3.4930	1.16434		
Plants	4	1.3733	0.34334	0.83	0.5078
Concentrations	5	0.9949	0.19898	0.48	0.7882
Plants* concentrations 20	1.2236	0.06118	0.15	1.0000	
Error	87	35.8564	0.41214		
Total	119	42.9412			

Grand Mean 49.91 CV 1.29

Table S8. Mean % mortality of *C. maculatus* after 24 h exposure period

Source	DF	SS	MS	F	P
Replication	3	453.3	151.11		
Plants	4	1195.0	298.75	10.62	0.0000
Concentration	5	8216.7	1643.33	58.43	0.0000
Plants*Concentration 20	475.0	23.75	0.84	0.6543	
Error	87	2446.7	28.12		
Total 119 12786.7					

Grand Mean 33.667 CV 15.75

Table S9. Mean % mortality of *C. maculatus* after 48 h exposure period

Source		DF	SS	MS	F		P		
Replication		3	943.3	314.44					
Plants		4	12538.3		3134.58		34.49	0.0000	
Concentration		5	14400.0		2880.00		31.69	0.0000	
Plants*Concentration	20	2341.7		117.08		1.29		0.2089	
Error		87	7906.7		90.88				
Total 119 38130.0									

Grand Mean 43.500 CV 21.92

Table S10. Mean % mortality of *C. maculatus* after 72 h exposure period

Source		DF	SS	MS	F		P		
Replication		3	251.2	83.72					
Plants		4	9976.3	2494.07		31.47	0.0000		
Concentration		5	10709.8		2141.97		27.02	0.0000	
Plants*Concentration	20	1320.7		66.03	0.83		0.6679		
Error		87	6895.6		79.26				
Total 119 29153.6									

Grand Mean 57.692 CV 15.43

Table S11. Mean % mortality of *C. maculatus* after 96 h exposure period

Source		DF	SS	MS	F		P		
Replication		3	871.3	290.42					
Plants		4	6929.2	1732.30		24.31	0.0000		
Concentration		5	13175.4		2635.07		36.98	0.0000	
Plants*Concentration	20	439.3	21.97	0.31		0.9979			
Error		87	6198.7		71.25				
Total 119 27613.9									

Grand Mean 77.967 CV 10.83

Table S12. Mean % repellency of *C. maculatus* after 1 h exposure period

Source		DF	SS	MS	F		P		
Replication		3	97.5	32.49					
Plants		4	19636.1		4909.02		13.29	0.0000	
Concentration		5	10892.2		2178.45		5.90		0.0001
Plants*Concentration	20	493.3	24.66	0.07		1.0000			
Error		87	32147.4		369.51				
Total 119 63266.5									

Grand Mean 39.168 CV 49.08

Table S13. Mean % repellency of *C. maculatus* after 6 h exposure period

Source		DF	SS	MS	F		P		
Replication		3	479.7	159.89					
Plants		4	22393.2		5598.30		36.16	0.0000	
Concentration		5	12768.9		2553.78		16.49	0.0000	
Plants*Concentration	20	1871.4		93.57	0.60		0.8996		
Error 87 13469.9 154.83									
Total 119 50983.1									

Grand Mean 43.857 CV 28.37

Table S14. Mean % repellency of *C. maculatus* after 12 h exposure period

Source		DF	SS	MS	F		P		
Replication		3	115.4	38.45					
Plants		4	21948.7		5487.17		12.31	0.0000	
Concentration		5	15086.7		3017.35		6.77		0.0000
Plants*Concentration	20	299.9	14.99	0.03		1.0000			
Error		87	38775.6		445.70				
Total 119 76226.2									

Grand Mean 47.684 CV 44.27

Table S15. Mean % repellency of *C. maculatus* after 24 h exposure period

Source		DF	SS	MS	F		P		
Replication		3	1882.8		627.61				
Plants		4	16740.7		4185.19		10.97		0.0000
Concentration		5	13899.1		2779.82		7.28		0.0000
Plants*Concentration	20	348.8	17.44	0.05		1.0000			
Error		87	33201.9		381.63				
Total 119 66073.4									

Grand Mean 59.528 CV 32.82

Table S16. Mean % repellency of *C. maculatus* after 48 h exposure period

Source		DF	SS	MS	F		P		
Replication		3	819.2	273.08					
Plants		4	20214.3		5053.58		13.75	0.0000	
Concentration		5	14494.6		2898.92		7.89		0.0000
Plants*Concentration	20	268.2	13.41	0.04		1.0000			
Error		87	31981.3		367.60				
Total 119 67777.6									

Grand Mean 65.093 CV 29.45

Table S17. Mean % repellency of *C. maculatus* after 72 h exposure period

Source		DF	SS	MS	F		P		
Replication		3	237.5	79.18					
Plants		4	8177.2	2044.30		7.36		0.0000	
Concentration		5	15762.7		3152.55		11.36	0.0000	
Plants*Concentration	20	609.7	30.49	0.11		1.0000			
Error		87	24149.5		277.58				
Total 119 48936.7									

Grand Mean 65.221 CV 25.55

Table S18. Mean % repellency of *C. maculatus* after 96 h exposure period

Source		DF	SS	MS	F		P		
Replication		3	1164.2		388.07				
Plants		4	11520.3		2880.07		8.89		0.0000
Concentration		5	13275.1		2655.02		8.19		0.0000
Plants*Concentration	20	329.8	16.49	0.05		1.0000			
Error		87	28196.2		324.09				
Total		119	54485.6						

Grand Mean 51.624 CV 34.87
